# The effect of marital and insurance status on the survival of elderly patients with stage M1b colon cancer: a SEER-based study

**DOI:** 10.1186/s12885-021-08627-5

**Published:** 2021-08-05

**Authors:** Chenghui Zhou, Yiwei Zhang, Xi Hu, Min Fang, Shuai Xiao

**Affiliations:** 1 Department of general surgery, Xiangya Hospital Central South University, Central South University, Changsha, China; 2 Department of General, Visceral, Cancer and Transplantation Surgery, University Hospital Cologne, Cologne, Germany; 3 Institute of Oncology, the First Affiliated Hospital, Hengyang Medical School, University of South, Hengyang, China; 4 Department of Gastrointestinal Surgery, the First Affiliated Hospital, Hengyang Medical School, University of South China, Hengyang, China

**Keywords:** Colon cancer, Marital status, Insurance status, overall survival, Cancer-specific survival

## Abstract

**Background:**

Colon cancer is largely implicated in elderly patients (age ≥ 60 years). The prognosis of patients diagnosed with the M1b stage is vastly poor. Marital and insurance status has been considered important prognostic factors in various cancer types. However, how these factors influence elderly patients with stage M1b colon cancer remains to be explored. This study aims to uncover the role of marital and insurance status in the survival of elderly patients with stage M1b colon cancer.

**Methods:**

We retrieved data for patients diagnosed with stage M1b colon cancer between 2010 and 2016 from the Surveillance, Epidemiology, and End Results (SEER) database. Our analysis of the clinicopathological features, overall survival (OS), and cancer-specific survival (CSS) was based on the marital and insurance status, respectively.

**Results:**

In sum, 5709 stage M1b colon cancer patients with complete information from SEER were enrolled for analysis. The OS and CSS of the Non-married group were poorer compared to that of the Married group. The OS and CSS of the Uninsured group were poorer than both of the Insured group and Medicaid group. However, OS was comparable between Uninsured group and Medicaid groups. The findings allude that marital and insurance status potentially impact the long-term survival of elderly patients with M1b colon cancer. The subgroup survival analyses revealed the lowest risk for death among the Insured Married group based on the comparison of the OS and CSS across all other groups. Moreover, Univariate and multivariate analyses revealed race, marital status, surgery, and chemotherapy as independent predictors for OS, whereas insurance status, surgery,and chemotherapy were independent predictors for CSS in elderly patients with M1b colon cancer.

**Conclusion:**

The marital and insurance status greatly impact the survival of elderly patients with M1b colon cancer. Therefore, it is imperative to provide more support to this vulnerable patient group who are lonely and uninsured, particularly in the psychological and health insurance aspect.

## Backgroud

Colon cancer is a common age-related diseases, considered one of the deadliest cancers worldwide [1]. It is well accepted that the incidence of colon cancer greatly increases with ageing and the median age at diagnosis is approximated at 70 years in developed countries [2]. According to the Surveillance, Epidemiology, and End Results (SEER) database (http://seer.cancer.gov/csr/1975_2017), over 45% of patients with stage IV colon cancer aged above 60 years are diagnosed at M1b stage [3]. Young adults and teenagers also may develop colon cancer but elderly patients exhibit significantly worse survival [4]. This echoes why elderly patients are an important component of the overall colon cancer entity, therefore, need to be given much focus.

These days, human beings have an increasingly longer life span. For the first time in history, an increasing percentage of people are expected to live into their sixties and beyond [5]. Considering the current situation, we may witness a significant increase in the number of elderly patients diagnosed with cancer. This will pose a serious social issue in the public health, and it is worthy of intense exploration [6].

The outcomes of cancer patients can be attributed to various factors, including clinicopathological factors, psychological factors, healthcare systems, and economic status [7]. Most previous studies focused on medical treatment but ignored the socioeconomic and health medical insurance factors. We propose the concept of sociomedical support, which incorporates both socioeconomic support and health medical insurance aids. Sociomedical support strongly impacts the survival of cancer patients [8]. Mounting evidence has demonstrated that social support provides important benefits to the emotional health of patients [9]. Marital status, as one of the fundamental social factors, has gradually attracted widespread attention [10–12]. Numerous studies have revealed that marital status potentially impacts the prognosis of various gastrointestinal cancer, including esophageal cancer [13], gastric cancer [14], and colorectal cancer [15]. Feng et al. found that married patients with colorectal signet-ring cell carcinoma were characterized with higher survival rates than Non-married patients [16]. Similarly, insurance status is associated with an increased survival rate in patients with colon cancer [17, 18].

It is notable that the overall physical status and disease resistance of the elderly are generally worse, and elderly patients have a lower overall survival rate than that of younger patients [19]. Cancer treatment is a huge challenge for young patients. Elderly patients with M1b colon cancer, however, will face more complex challenges, in particular, those lacking social and family support. Patients with resectable colon cancer receive short-term therapy and benifit from a high efficiency-cost ratio of treatment. As Such, sociomeidical support may have relatively less impact on these patients. However, sociomeidical support may significantly impact the prognosis of M1b colon cancer in elderly patients. Their prognosis may worsen due to the lack of family care and economic support,.

This study explores how marital and insurance status impact the survival of elderly patients with M1b colon cancer through analysis of data from the Surveillance Epidemiology and End Results (SEER) database.

## Methods

### Data source

We retrieved data from the SEER cancer registry, an open and reliable database that provides demographic, epidemiological, and following-up information. Cases from 18 SEER registries in the anonymous data were analyzed. We acquired permission to download the data from the SEER database, which did not require informed patient consent.

### Patient selection

**Fig. 1:**
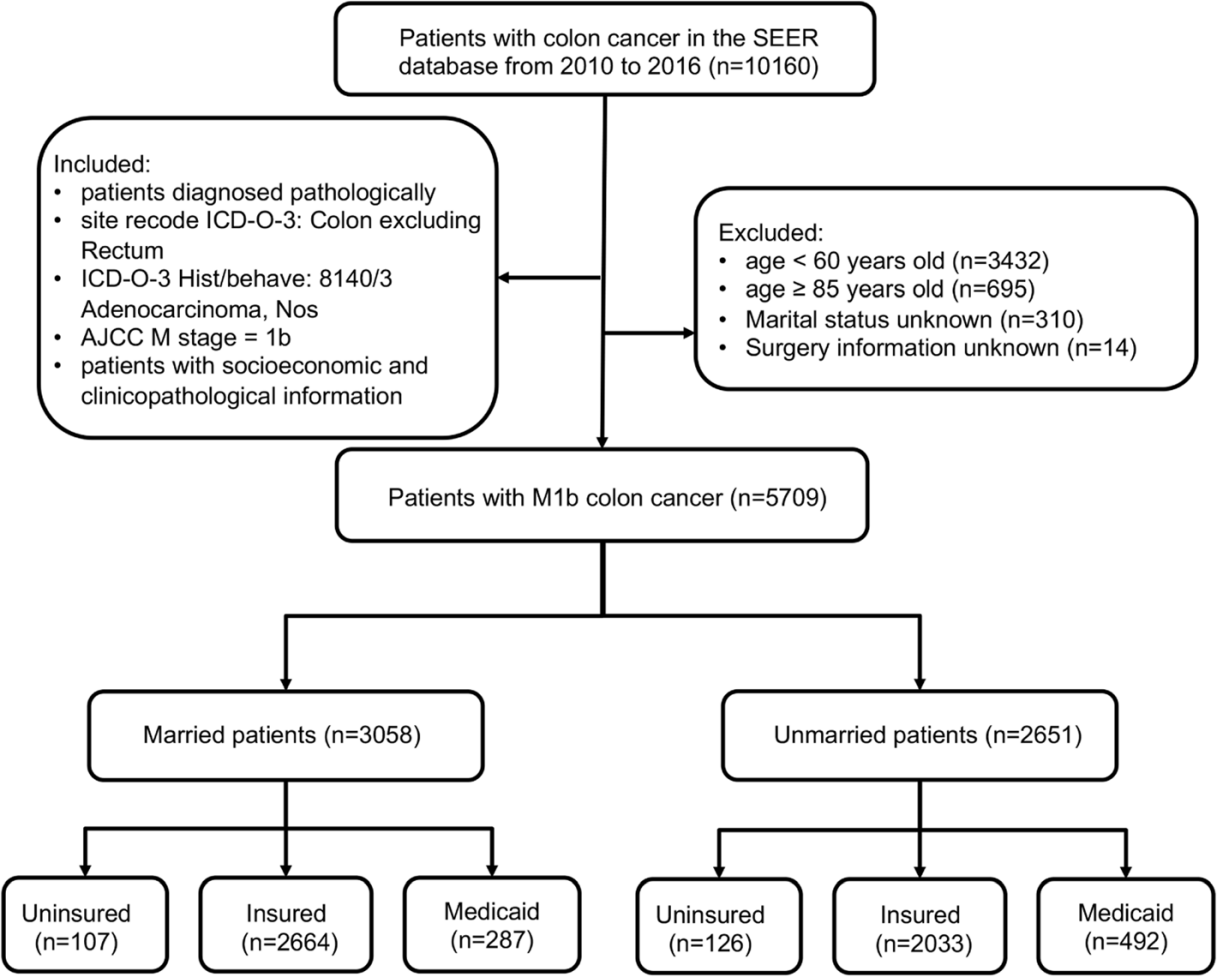
Flowchart of patient inclusion and exclusion into the study. SEER: Surveillance, Epidemiology, and End Results; AJCC: Americal Americal Joint Committee on Cancer

We explored the SEER database using the SEER software (SEER*Stat 8.3.9, Released March 15), and enrolled patients diagnosed with M1b colon cancer between 2010 and 2016 (Fig. 1). All cases were coded according to the International Classification of Diseases for Oncology (ICD-O-3). The inclusion for colon cancer patients were as following: 1) patients were pathologically confirmed; 2) site recode ICD-O-3: Colon excluding Rectum; 3) ICD-O-3 Hist/behave: 8140/3 Adenocarcinoma, Nos; 4) American Joint Committee on Cancer (AJCC) 7th ed. M stage = 1b; 5) patients with marital and insurance status and clinicopathological information in the database. The exclusion criteria were as follows: 1) patients age < 60 or age ≥ 85 years old at diagnosis; 2) the marital status was unclear; 3) unknown insurance recode; 4) patients without surgery information. The other baseline data were extracted for all patients in the SEER database, including age, sex, race, surgery, radiotherapy, chemotherapy, marital, and health insurance status.

### Description of the key variables

The study variables included age, sex, race, surgery, radiotherapy and chemotherapy, marital status and insurance status. Marital status was defined as Married (including cohabited/partner as well as married) and Non-married (never married, divorced, separated, or widowed), whereas insurance status was defined as Insured, Uninsured and Medicaid.

### Statistical analysis

Descriptive statistics of patient characteristics were summarized. After that, we compared the socioeconomic status, clinicopathological characteristics of M1b colon cancer patients using the chi-square test (χ2). For each patient, the survival outcomes analyzed were as follows: 1) overall survival (OS), defined as the time from the date of diagnosis to death from any cause; 2) cancer-specific survival (CSS), defined as the time from the date of diagnosis until cancer metastasis or recurrence, cancer-associated death and the end of follow-up. Both 5- year OS and 5- year CSS were estimated using Kaplan–Meier survival curves. The log-rank test was applied to compare the differences among these groups. The prognostic factors associated with OS and CSS were subjected to univariate and multivariable Cox proportional regression analyses.All statistical data were analyzed with the software package SPSS version 22.0 (SPSS Inc., Chicago, IL, USA). A*P-*value < 0.05 denoted statistical significance.

## Results

### General characteristics of elderly M1b Colon Cancer patients according to marital status and insurance status

We enrolled 5709 cases reported between 2010 and 2016 retrieved from the SEER database according to the inclusion and exclusion criteria. The baseline demographic, clinicopathological, and surgery features were analyzed and compared in Tables 1 and 2, respectively. Results in Table 1 shows 46.4% (*n* = 2651) were Non-married patients, 53.6% (*n* = 3058) patients were Married. The median survival time of the enrolled patients was 7 months, with11.74 ± 13.55 months as the average survival time. Differences in demographic and insurance status and therapy characteristics between the Married group and the Non-married group were statistically significant in terms of sex, race, surgery, radiotherapy, chemotherapy, and insurance status (*P* < 0.05). Results in Table 2 shows 4.3% (*n* = 233) were Uninsured patients, 82.6% (*n* = 4697) patients were Insured, 13.1% (*n* = 779) were Medicaid. We found significant differences in demographic and insurance status and therapy characteristics among these groups in terms of race, surgery, chemotherapy, and marital status (*P* < 0.001). However, sex and radiotherapy were not significantly different between these groups (*P* > 0.1).

**Table 1:** Characteristics of elderly patients with M1b colon cancer by marital status

	Total	Single/Widowed/Separated/Divorced (2651)	Married/Partner (3058)	*P*/Value
**Sex**				0.000
Male	2970	1058 (39.9%)	1912 (62.5%)	
Female	2739	1593 (60.1%)	1146 (37.5%)	
**Race**				0.000
White	4373	1932 (72.9%)	2441 (79.8%)	
Black	855	538 (20.3%)	317 (10.4%)	
Others	481	181 (6.8%)	300 (9.8%)	
**Surgery**				0.001
None	3106	1504 (56.7%)	1602 (52.4%)	
Surgery	2603	1147 (43.3%)	1456 (47.6%)	
**Radiotherapy**				0.016
None/Unknown	5444	2547 (96.1%)	2897 (94.7%)	
Yes	265	104 (3.9%)	161 (5.3%)	
**Chemotherapy**				0.000
None/Unknown	2428	1316 (49.6%)	1112 (36.4%)	
Yes	3281	1335 (50.4%)	1946 (63.6%)	
**Insurance status**				0.000
Uninsured	233	126 (4.8%)	107 (3.5%)	
Insured	4697	2033 (76.7%)	2664 (87.1%)	
Medicaid	779	492 (18.6%)	287 (9.4%)

**Table 2:** Characteristics of elderly patients with M1b colon cancer by insurance status

	Total	Uninsured (233)	Insured (4697)	Medicaid (779)	*P*/Value
**Sex**					0.901
Male	2970	123 (52.8%)	2447 (52.1%)	400 (51.3%)	
Female	2739	110 (47.2%)	2250 (47.9%)	379 (48.7%)	
**Race**					0.000
White	4373	169 (72.5%)	3731 (79.4%)	473 (60.7%)	
Black	855	51 (21.9%)	628 (13.4%)	176 (22.6%)	
Others	481	13 (5.6%)	338 (7.2%)	130 (16.7%)	
**Surgery**					0.018
None	3106	138 (59.2%)	2515 (53.5%)	453 (58.2%)	
Surgery	2603	95 (40.8%)	2182 (46.5%)	326 (41.8%)	
**Radiation**					0.172
None/Unknown	5444	221 (94.8%)	4470 (95.2%)	753 (96.7%)	
Yes	265	12 (5.2%)	227 (4.8%)	26 (3.3%)	
**Chemotherapy**					0.000
None/Unknown	2428	112 (48.1%)	1919 (40.9%)	397 (51.0%)	
Yes	3281	121 (51.9%)	2778 (59.1%)	382 (49.0%)	
**Marital status**					0.000
Single/Widowed/Separated/Divorced	2651	126 (54.1%)	2033 (43.3%)	492 (63.2%)	
Married/Partner	3058	107 (45.9%)	2664 (56.7%)	287 (36.8%)	

Moreover, the Married group constituted a higher proportion of male or white patients, treated patients (surgery, radiotherapy and chemotherapy), and insured patients than the Non-married group (*P* < 0.05). The highest percentages of patients in the insured group included the white race, patients treated with surgery or chemotherapy, and by Married patients, which significantly differed from the other insurance status groups (P < 0.05). However, sex and radiation treatment were similar among these groups (both *P* > 0.05).

### Long-term survival of Colon Cancer according to marital status and insurance status

**Fig. 2:**
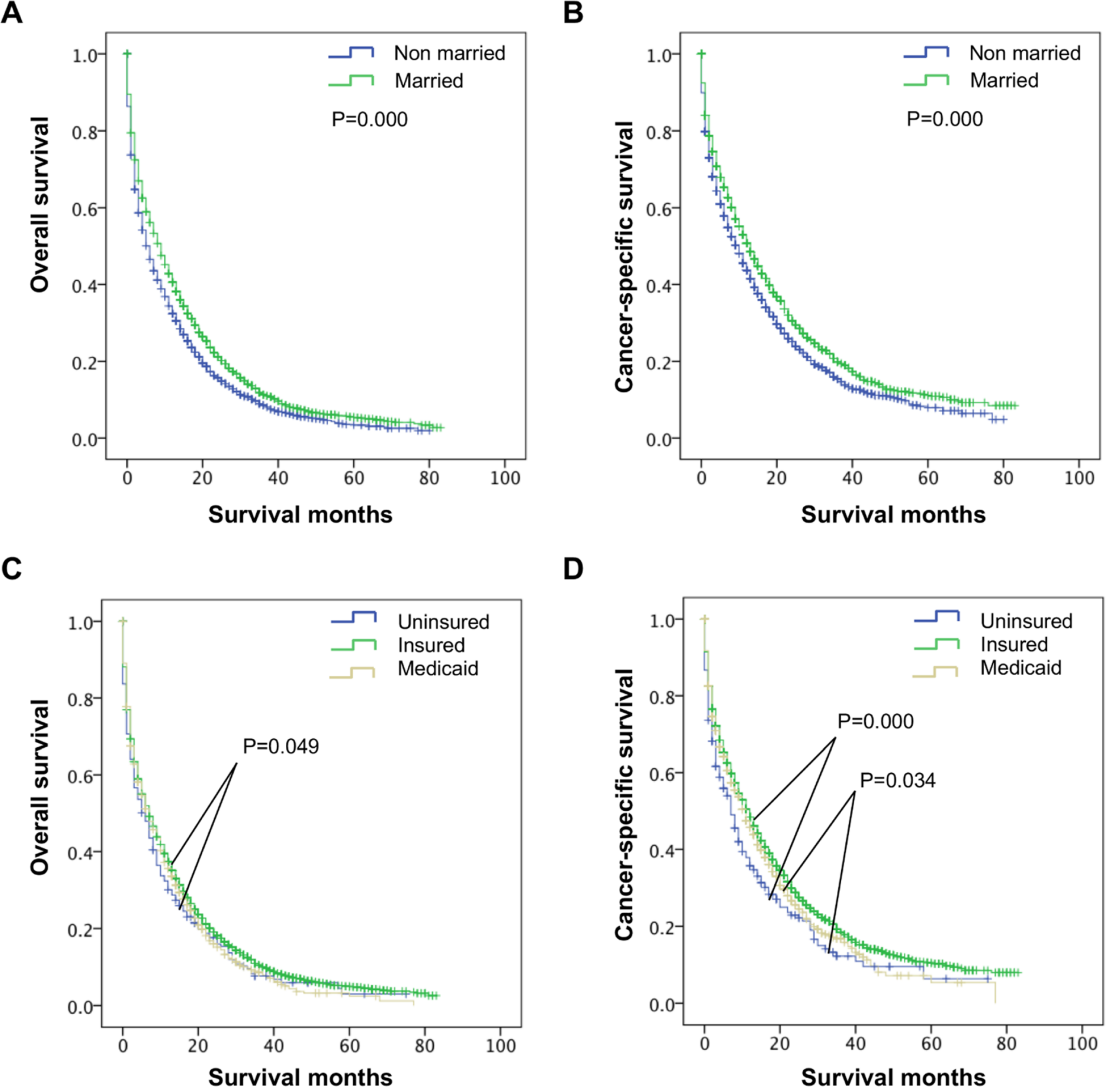
Long-term survival of of elderly M1b colon cancer patients according to marital status and insurance status, respectively. **A**, **B** the survival curves showed that the OS and CSS of the Non-married group were poorer than that of the Married group **C**, **D** the survival curves showed that the OS and CSS of the Uninsured group was poorer than that of the Insured group. **C** the Medicaid group had the similar OS with other subgroups. **D** the Medicaid group had the similar CSS with Insured group but had a better CSS than Uninsured group. OS, overall survival; CSS, cancer-specific survival

To explore whether married status and insurance status had benefits for long-term survival, we evaluated the potential survival difference between these patients via Kaplan-Meier analysis and log-rank tests. The results revealed pooper OS and CSS of the Non-married group than that of the Married group (*P* = 0.000, Fig. 2A and B). The OS of the Uninsured group was poorer compared to that of the Insured group (*P* = 0.049, Fig. 2C), but with similar OS between Uninsured group and Medicaid group, Medicaid group and Insured group (*P* > 0.05, Fig. 2C). Furthermore, the CSS of the Uninsured group was poorer than that of the Insured (*P* > 0.00, Fig. 2D) or Medicaid groups (*P* > 0.028, Fig. 2D). Accordingly, the Insured group had the best CSS (20.72 ± 0.465 months), followed by the Medicaid group (17.68 ± 0.996 months) and the Uninsured group (15.55 ± 1.627 months) (P > 0.05, Fig. 2C). These data demonstrate that marital status and insurance status potentially have an impact on the long-term survival of elderly patients with M1b colon cancer.

### The impact of the interplay of marital status and insurance status on Long-term survival

**Fig. 3:**
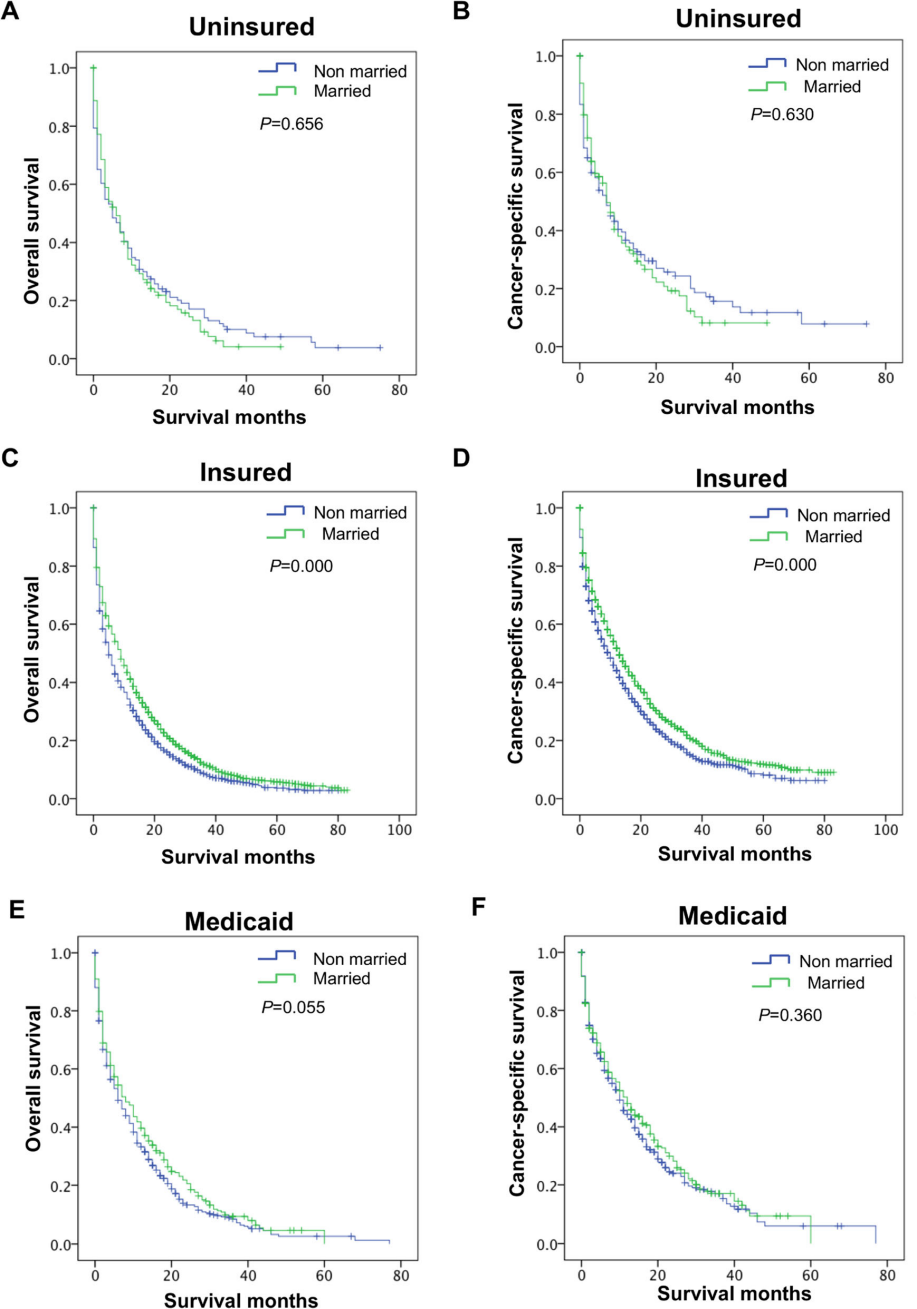
Long-term survival of elderly M1b colon cancer patients grouped by marital status stratified by insurance status. **A**, **B** The stratified analysis survival curves showed that the Married group who are uninsured had comparable OS **A** and CSS **B** with the Unmarried of group; **C**, **D** The stratified analysis survival curves showed that the Married group who are insured had better OS **C** and CSS **D** with the Unmarried group. **E**, **F** The stratified analysis survival curves showed that the Married group who recevied medicaid had comparable OS **E** and CSS **F** with the Unmarried group. OS, overall survival; CSS, cancer-specific survival

We previously found lower OS and CSS among the Non-married group compared to the Married group. Herein, the insured group had better OS and CSS than the uninsured group. Further analysis of the survival differences between different marital statuses via stratification of insurance statuses revealed that when insured, the OS and CSS of the Non-married group were both worse than that of the Married group (*P* = 0.000, Fig. 3C and D). In other insurance statuses, that are, the uninsured group and Medicaid group, the OS and CSS of the Married group were similar to the Unmarried group (*P* > 0.05, Fig. 3A and B, E and F).

**Fig. 4:**
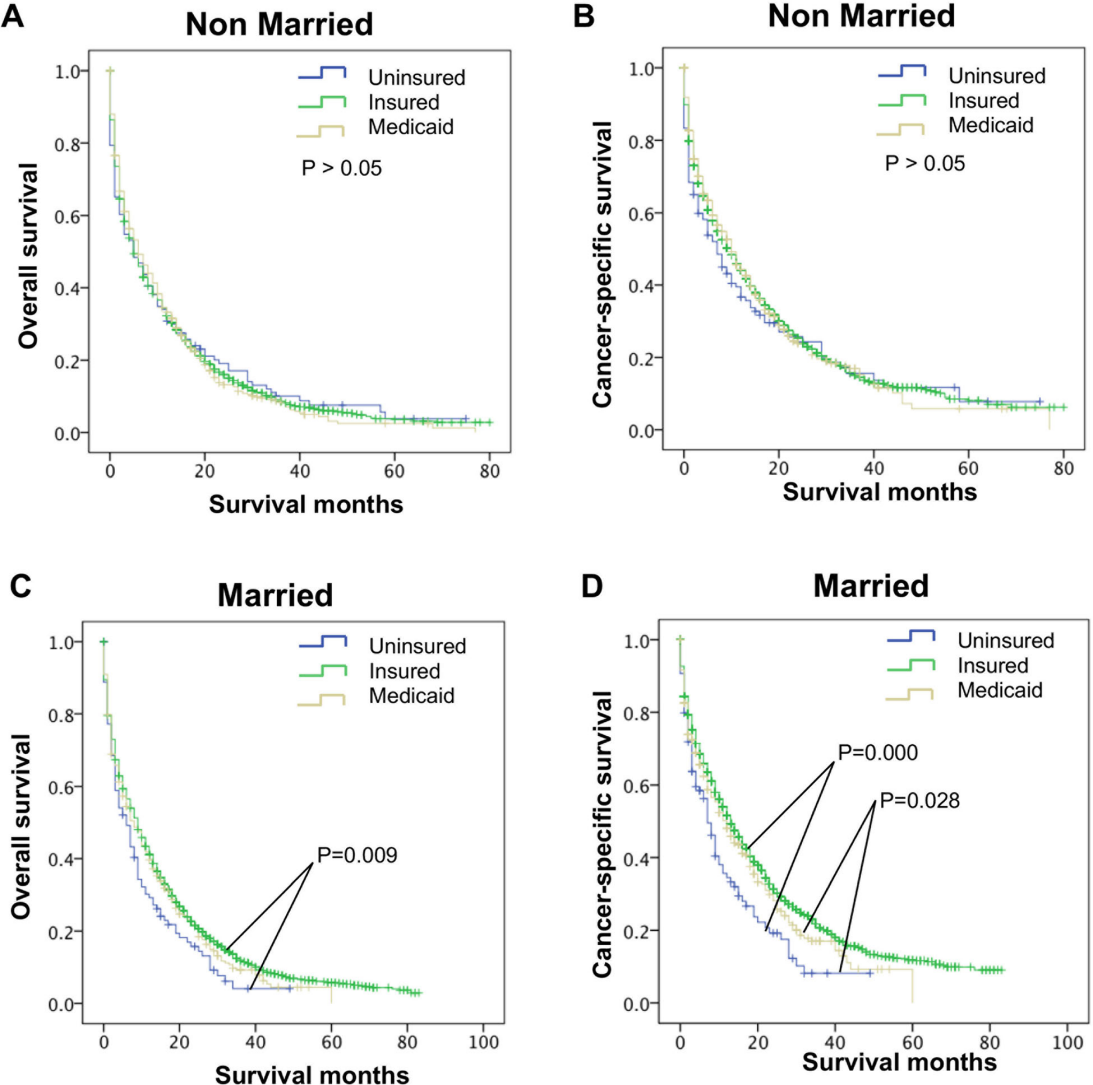
Long-term survival of elderly M1b colon cancer patients grouped by insurance status stratified by marital status. **A**, **B** The stratified analysis survival curves showed that when in Unmarried group, OS **A** and CSS **B** were both comparable with those Uninsured, Insured and Medicaid group; **C**, **D** The stratified analysis survival curves showed that when in Married group, the OS **C** and CSS **D** of the Uninsured group were both worse than those Insured group, and the OS of the Uninsured group had similar OS **C** but worse CSS **D** to those Medicaid group

Further evaluation of the impact of insurance statuses on OS and CSS rates for the Non-married and Married group showed that for Unmarried patients, their OS and CSS were both comparable with those in the Uninsured, Insured, and Medicaid groups (P > 0.05, Fig. 4A and B). But in the context of Married, the OS (*P* = 0.009, Fig. 4C) and CSS (P = 0.000, Fig. 4D) of the Uninsured group were both worse than those the Insured group. Moreover, the OS of the Uninsured group was similar to those Medicaid group (P > 0.05, Fig. 4C), whereas the CSS of the Uninsured group was worse than those Medicaid group (*P* = 0.028, Fig. 4D). These data demonstrate that married patients with insurance are associated with the lowest risk of death when the OS and CSS are compared among all the groups.

### Risk factors for Long-term survival of elderly M1b Colon Cancer

The risk factors for survival of elderly patients with M1b colon cancer were explored via univariate or multivariable analyses (Tables 3 and 4). The results implicated that race, insurance status, marital status, surgery and chemotherapy as significant prognostic factors for OS in elderly M1b colon cancer patients in univariate analyses (*P* < 0.05). At the same time, other factors, including race (Asian, Latino), married, surgery and chemotherapy were associated with better OS. Such as association remained significant in multivariable analyses that excluded insurance status (*P* = 0.068). However, sex and radiation therapy were not significantly associated with OS in elderly M1b colon cancer patients in both univariate and multivariable analyses (both *P* > 0.05, Table 3). Of note, race, married, surgery and chemotherapy were revealed as significant independent prognostic factors for OS in elderly M1b colon cancer patients.

**Table 3:** Univariate and multivariable analysis of factors associated with overall survival of elderly M1b colon cancer patients

Variable	Univariate		Multivariable	
	HR(95%CL)	*P*	HR(95%CL)	*P*
**Sex**				
Male	1		1	
Female	0.979 (0.927–1.035)	0.463	0.983 (0.929–1.041)	0.560
**Race**				
White	1	0.009	1	0.001
Black	1.033 (0.956–1.116)	0.415	0.982 (0.907–1.063)	0.650
Others	0.863 (0.779–0.956)	0.005	0.822 (0.741–0.912)	0.000
**Insurance status**				
Uninsured	1	0.045	1	0.068
Insured	0.873 (0.759–1.004)	0.057	0.946 (0.822–1.089)	0.440
Medicaid	0.938 (0.803–1.096)	0.423	0.865 (0.740–1.012)	0.070
**Marital status**				
Single/Widowed/Separated/Divorced	1		1	
Married/Partner	0.828 (0.783–0.875)	0.000	0.940 (0.886–0.996)	0.037
**Surgery**				
None	1		1	
Surgery	0.547 (0.516–0.579)	0.000	0.499 (0.471–0.529)	0.000
**Radiation**				
None/Unknown	1		1	
Yes	1.075 (0.945–1.222)	0.274	1.029 (0.904–1.171)	0.666
**Chemotherapy**				
None/Unknown	1		1	
Yes	0.366 (0.345–0.387)	0.000	0.341 (0.321–0.361)	0.000

**Table 4:** Univariate and multivariable analysis of factors associated with cancer-specific survival of elderly M1b colon cancer patients

Variable	Univariate		Multivariable	
	HR(95%CL)	*P*	HR(95%CL)	*P*
**Sex**				
Male	1		1	
Female	0.985 (0.924–1.050)	0.635	0.992 (0.929–1.059)	0.810
**Race**				
White	1	0.131	1	0.124
Black	1.068 (0.977–1.167)	0.146	1.011 (0.924–1.107)	0.809
Others	0.933 (0.831–1.046)	0.233	0.889 (0.791–0.999)	0.048
**Insurance status**				
Uninsured	1	0.001	1	0.010
Insured	0.768 (0.660–0.895)	0.001	0.832 (0.714–0.970)	0.018
Medicaid	0.837 (0.705–0.993)	0.041	0.767 (0.645–0.911)	0.002
**Marital status**				
Single/Widowed/Separated/Divorced	1		1	
Married/Partner	0.833 (0.782–0.888)	0.000	0.945 (0.884–1.010)	0.097
**Surgery**				
None	1		1	
Surgery	0.531 (0.497–0.567)	0.000	0.488 (0.456–0.522)	0.000
**Radiation**				
None/Unknown	1		1	
Yes	1.086 (0.937–1.259)	0.274	1.036 (0.893–1.201)	0.640
**Chemotherapy**				
None/Unknown	1		1	
Yes	0.384 (0.360–0.410)	0.000	0.358 (0.334–0.383)	0.000

In addition, univariate analyses revealed insurance status, marital status, surgery, and chemotherapy were significant prognostic factors for CSS in elderly M1b colon cancer (P < 0.05). Being married, insured, or receiving support from Medicaid, or having surgery and chemotherapy were associated with better OS. Such association remained significant in multivariable analyses that excluded marital status (*P* = 0.097). Notably, sex and radiotherapy were not significantly associated with CSS in colon cancer in both univariate and multivariable analyses (both P > 0.05, Table 4). Based on the results, being insured, having surgery, and chemotherapy were significant favorable independent prognostic factors for CSS in elderly M1b colon cancer patients.

## Discussion

There is evidence that marital and insurance status provide survival benefits in gastrointestinal cancers, including esophageal cancer [13], gastric cancer [14], and colorectal cancer [15]. However, researchers are yet to explore the survival of elderly patients with M1b colon cancer whose tumor cannot undergo radical resection. Elderly patients with M1b colon cancer generally receive long-term therapy and suffer from the low efficiency-cost ratio of their treatment. The majority of these patients are lonely and lack the funds to manage medical conditions. Psychological support and health insurance may greatly influence these disadvantaged groups. Therefore, it is imperative to evaluate whether marital and insurance status potentially impacts the prognosis of elderly patients with M1b colon cancer.

In this study, we found that sociomedical support, including marital and insurance status, significantly impacted the survival of elderly M1b colon cancer patients. Subgroup analysis revealed that insured married patients had the best OS and CSS compared to other subgroups. However, for the uninsured group, patients exhibited similar results for OS and CSS regardless of their marital status. Likewise, for the Non-married group, patients exhibited similar results for OS and CSS regardless of their insurance status. Further analysis implicated the status of being married and having health insurance as the independent prognostic factors for elderly patients with M1b colon cancer.

With the advent of an aging society, the proportion of elderly people is seeing an unprecedented rise in several nations, which is indeed a global societal threat [20]. It is also a unique challenge for lonely persons diagnosed with M1b colon cancer, particularly for those elderly patients without insurance support [21]. Sociomedical support improves the mental health and survival of patients diagnosed with cancer [22, 23]. Previous reports have confirmed that marital and insurance status are associated with the clinical outcome of colon cancer, and the subject patients in these studies mainly those who had resectable colon cancer and were scattered into different age groups [15, 18, 24]. However, the relationship between marital and insurance status and M1b colon cancer remains to be explored, especially in elderly patients aged above 60 years. Our study has demonstrated that marital and insurance statuses were independent protective prognostic factors in elderly patients with M1b colon cancer. Also, the OS and CSS of married patients were remarkably better than those of non-married patients, whereas insured patients had better CSS and comparable OS compared to those uninsured. We further revealed that, for the insured group, OS and CSS of the non-married were worse than the married patients. Similarly, for the married group, the OS and CSS of the uninsured were worse than the insured.These data suggest the need for more evidence-based studies on the socioeconomic support and health insurance plans for elderly patients with cancer.

The following potential mechanisms may explain the findings above. Firstly, married and the insured patients have better adherence compared to the non-married patients; they have a higher tendency to attend regular medical appointments and health checkup procedures and follow the essential medical care and treatment timely [25]. It is consistent with our results that the married group and the insured group had the highest proportion of treated patients (i.e., surgery, radiotherapy, and chemotherapy) than other subgroups. Numerous studies have revealed that surgery, radiotherapy, and chemotherapy improve the outcome of colon cancer patients, which may explain the better prognosis reported in the married group [26, 27]. Secondly, the married have more stable financial sources compared to the non-married. For example, they can obtain financial support from their spouses or partners to cover the health insurance and medical expenses or share the same insurance account with their spouses. We found the married group accounted for the highest proportion of insured patients than patients from other subgroups. It was also noted that the insured group accounted for the highest proportion of patients who had received surgery and chemotherapy treatment than patients from other subgroups. Thirdly, owing to a lack of psychological support, non-married patients might suffer more depression, anxiety, and stress than married patients after they are diagnosed with cancers [28]. These psychological effects could trigger immune interactions and endocrine hormones, which promote tumor progression, and influence treatment effects and outcomes of patients [29–32]. Numerous studies have described the effect of stress on immune response [33, 34], for instance, Hamilton et al. found that elevated inflammatory factors were associated with worse survival of colon cancer patients [35]. Elsewhere, Miller et al. revealed that chronic psychological stress impaired immune response and changed hormones level [36]. Particularly, cortisol, a stress hormones, would be released in high doses under psychological stress, promoting cancer metastasis and negatively impact on response to cancer treatment [32, 37, 38]. It is worth noting that psychological stress appears to influence the functions of an organ and system functions at multiple levels, and more experimental evidence is warranted in this area [39]. These data indicate a close association of sociomedical support with patient prognosis, especially for elderly patients with cancer. A lack of emotional support and medical insurance means that this category of cancer patients would miss out on effective treatment and have a poor prognosis.

Collectively, this study found marital and insurance status plays a crucial role in improving the survival of elderly patients with M1b colon cancer. However, there are some limitations to the study. First and foremost, marital status was constrained to legal marital status; thus, non-married individuals cohabiting with a partner were registered as single individuals and could be categorized as unmarried. Such patients would be expected to have a better prognosis than the unmarried, which may introduce bias to the survival results. Secondly, marital status was recorded at diagnosis, therefore, whether the marital status of patients changed throughout the follow-up period remains unknown. Thirdly, although the SEER database provided data on marital status, the quality of marriage remains unknown. As such,we could not precisely identify the psychological support for survival. Finally, data on socioeconomic factors such as income, residence, education level and employment status, and mental disease are not available in the SEER database, yet these factors relate to and influence one another. For instance, the income of an individual determines the insurance type, and education level correlates with their income. Therefore, such disparities among diferent marital statuses should be investigated in future studies.and education level correlates with their income. Therefore, such disparities among diferent marital statuses should be investigated in future studies.and education level correlates with their income. Therefore, such disparities among diferent marital statuses should be investigated in future studies.

In conclusion, this study highlights the substantial impacts of marital and insurance status on the survival of elderly patients with M1b colon cancer. Compared to married patients, non-married patients suffer a higher risk of death. This raises awareness that psychological support for vulnerable populations, especially elderly M1b colon cancer patients without marriage and insurance, could substantially improve their long-term survival rate.

## Data Availability

Publicly available datasets were analyzed in this study. These data can be found here: Surveillance, Epidemiology, and End Results (SEER) database (https://seer.cancer.gov/).

## Abbreviations

AJCC: Americal Joint Committee on Cancer
THE: Carcinoembryonic antigen
CSS: Cancer-specific survival
ICD-O: International Classification of Diseases for Oncology
YOU: Overall survival
WHO: World Health Organization
SEER: Surveillance, Epidemiology, and End Results

## References

[1] Siegel RL, Miller KD, Goding Sauer A, Fedewa SA, Butterly LF, Anderson JC, et al. Colorectal cancer statistics, 2020. CA Cancer J Clin. 2020;70(3):145–64. 10.3322/caac.21601 32133645

[2] Brenner H, Kloor M, Pox CP. Colorectal cancer. Lancet. 2014;383(9927):1490–502. 10.1016/S0140-6736(13)61649-9 10.1016/S0140-6736(13)61649-9 24225001

[3] Jung KW, Won YJ, Kong HJ, Oh CM, Lee DH, Lee JS. Prediction of cancer incidence and mortality in Korea, 2014. Cancer Res Treat. 2014;46(2):124–30. 10.4143/crt.2014.46.2.124. 10.4143/crt.2014.46.2.124 PMC4022820 24851103

[4] Chandrasinghe PC, Ediriweera DS, Nazar T, Kumarage S, Hewavisenthi J, Deen KI (2017). Overall survival of elderly patients having surgery for colorectal Cancer is comparable to younger patients: results from a south Asian population. Gastroenterol Res Pract.

[5] Beard JR, Officer A, de Carvalho IA, Sadana R, Pot AM, Michel JP, et al. The world report on ageing and health: a policy framework for healthy ageing. Lancet. 2016;387(10033):2145–54. 10.1016/S0140-6736(15)00516-4. 10.1016/S0140-6736(15)00516-4 PMC4848186 26520231

[6] Carioli G, Malvezzi M, Bertuccio P, Hashim D, Waxman S, Negri E, et al. Cancer mortality in the elderly in 11 countries worldwide, 1970-2015. Ann Oncol. 2019;30(8):1344–55. 10.1093/annonc/mdz178. 10.1093/annonc/mdz178 31147682

[7] Kivimäki M, Batty GD, Pentti J, Shipley MJ, Sipilä PN, Nyberg ST, et al. Association between socioeconomic status and the development of mental and physical health conditions in adulthood: a multi-cohort study. Lancet Public Health. 2020;5(3):e140–9. 10.1016/S2468-2667(19)30248-8 10.1016/S2468-2667(19)30248-8 32007134

[8] Arnold M, Rutherford MJ, Bardot A, Ferlay J, Andersson TM, Myklebust T, et al. Progress in cancer survival, mortality, and incidence in seven high-income countries 1995-2014 (ICBP SURVMARK-2): a population-based study. Lancet Oncol. 2019;20(11):1493–505. 10.1016/S1470-2045(19)30456-5 10.1016/S1470-2045(19)30456-5 PMC6838671 31521509

[9] Holt-Lunstad J (2018). Why social relationships are important for physical health: a systems approach to understanding and modifying risk and protection. Annu Rev Psychol.

[10] Shapiro M, Chen Q, Huang Q, Boosalis VA, Yoon CH, Saund MS, et al. Associations of socioeconomic variables with resection, stage, and survival in patients with early-stage pancreatic Cancer. JAMA Surg. 2016;151(4):338–45. 10.1001/jamasurg.2015.4239 10.1001/jamasurg.2015.4239 26581025

[11] Whisman MA, Gilmour AL, Salinger JM (2018). Marital satisfaction and mortality in the United States adult population. Health Psychol.

[12] Wong CW, Kwok CS, Narain A, Gulati M, Mihalidou AS, Wu P, et al. Marital status and risk of cardiovascular diseases: a systematic review and meta-analysis. Heart. 2018;104(23):1937–48. 10.1136/heartjnl-2018-313005 10.1136/heartjnl-2018-313005 29921571

[13] He Y, Liang D, Du L, Guo T, Liu Y, Sun X, et al. Clinical characteristics and survival of 5283 esophageal cancer patients: a multicenter study from eighteen hospitals across six regions in China. Cancer Commun (Lond). 2020;40(10):531–44. 10.1002/cac2.12087 10.1002/cac2.12087 PMC7571391 32845581

[14] Zhou R, Yan S, Li J (2016). Influence of marital status on the survival of patients with gastric cancer. J Gastroenterol Hepatol.

[15] Yang CC, Cheng LC, Lin YW, Wang SC, Ke TM, Huang CI, et al. The impact of marital status on survival in patients with surgically treated colon cancer. Medicine (Baltimore). 2019;98(11):e14856. 10.1097/MD.0000000000014856 10.1097/MD.0000000000014856 PMC6426559 30882684

[16] Feng L, Yang YJ, Du J, Yu YJ, Diao JD (2020). Marital status and survival of patients with colorectal signet ring cell carcinoma: a population-based study. Sci Rep.

[17] Ratjen I, Schafmayer C, Enderle J, di Giuseppe R, Waniek S, Koch M, et al. Health-related quality of life in long-term survivors of colorectal cancer and its association with all-cause mortality: a German cohort study. BMC Cancer. 2018;18(1):1156. 10.1186/s12885-018-5075-1 10.1186/s12885-018-5075-1 PMC6251222 30466408

[18] Sun W, Cheng M, Zhuang S, Qiu Z (2019). Impact of insurance status on stage, treatment, and survival in patients with colorectal Cancer: a population-based analysis. Med Sci Monit.

[19] Quaglia A, Tavilla A, Shack L, Brenner H, Janssen-Heijnen M, Allemani C, et al. The cancer survival gap between elderly and middle-aged patients in Europe is widening. Eur J Cancer. 2009;45(6):1006–16. 10.1016/j.ejca.2008.11.028 10.1016/j.ejca.2008.11.028 19121578

[20] Partridge L, Deelen J, Slagboom PE (2018). Facing up to the global challenges of ageing. Nature.

[21] Keum N, Giovannucci E. Global burden of colorectal cancer: emerging trends, risk factors and prevention strategies. Nat Rev Gastroenterol Hepatol. 2019;16(12):713–32 .10.1038/s41575-019-0189-8 10.1038/s41575-019-0189-8 31455888

[22] Lin D, Gold HT, Schreiber D, Leichman LP, Sherman SE, Becker DJ (2018). Impact of socioeconomic status on survival for patients with anal cancer. Cancer.

[23] Liu L, Chi YY, Wang AA, Luo Y (2018). Marital status and survival of patients with hormone receptor-positive male breast Cancer: a surveillance, epidemiology, and end results (SEER) population-based study. Med Sci Monit.

[24] Xiao K, Zhao Y, Cai Y, Chen P, Chen J, Ye R, et al. The effect of marital status on the survival of patients with colorectal neuroendocrine neoplasms: an analysis of the SEER database. Rev Esp Enferm Dig. 2020;112(2):109–17.10.17235/reed.2019.6183/2019 10.17235/reed.2019.6183/2019 31830797

[25] Pietrzykowski Ł, Michalski P, Kosobucka A, Kasprzak M, Fabiszak T, Stolarek W, et al. Medication adherence and its determinants in patients after myocardial infarction. Sci Rep. 2020;10(1):12028.10.1038/s41598-020-68915-1 10.1038/s41598-020-68915-1 PMC7374107 32694522

[26] van Steenbergen LN, Elferink MAG, Krijnen P, Lemmens V, Siesling S, Rutten HJT, et al. Improved survival of colon cancer due to improved treatment and detection: a nationwide population-based study in the Netherlands 1989-2006. Ann Oncol. 2010;21(11):2206–12. 10.1093/annonc/mdq227 10.1093/annonc/mdq227 20439339

[27] Aizer AA, Chen MH, McCarthy EP, Mendu ML, Koo S, Wilhite TJ, et al. Marital status and survival in patients with cancer. J Clin Oncol. 2013;31(31):3869–76. 10.1200/JCO.2013.49.6489 10.1200/JCO.2013.49.6489 PMC4878087 24062405

[28] Pitman A, Suleman S, Hyde N, Hodgkiss A (2018). Depression and anxiety in patients with cancer. Bmj.

[29] Antoni MH, Dhabhar FS (2019). The impact of psychosocial stress and stress management on immune responses in patients with cancer. Cancer.

[30] Schakel L, Veldhuijzen DS, Crompvoets PI, Bosch JA, Cohen S, van Middendorp H, et al. Effectiveness of stress-reducing interventions on the response to challenges to the immune system: a meta-analytic review. Psychother Psychosom. 2019;88(5):274–86. 10.1159/000501645 10.1159/000501645 PMC6878733 31387109

[31] Wynne B, McHugh L, Gao W, Keegan D, Byrne K, Rowan C, et al. Acceptance and Commitment Therapy Reduces Psychological Stress in Patients With Inflammatory Bowel Diseases. Gastroenterology. 2019;156(4):935–45 e931. 10.1053/j.gastro.2018.11.030 30452919

[32] Russell G, Lightman S (2019). The human stress response. Nat Rev Endocrinol.

[33] Yang H, Xia L, Chen J, Zhang S, Martin V, Li Q, et al. Stress-glucocorticoid-TSC22D3 axis compromises therapy-induced antitumor immunity. Nat Med. 2019;25(9):1428–41.10.1038/s41591-019-0566-4 10.1038/s41591-019-0566-4 31501614

[34] Xu C, Lee SK, Zhang D, Frenette PS (2020). The Gut Microbiome Regulates Psychological-Stress-Induced Inflammation. Immunity.

[35] Hamilton TD, Leugner D, Kopciuk K, Dixon E, Sutherland FR, Bathe OF (2014). Identification of prognostic inflammatory factors in colorectal liver metastases. BMC Cancer.

[36] Miller GE, Cohen S, Ritchey AK (2002). Chronic psychological stress and the regulation of pro-inflammatory cytokines: a glucocorticoid-resistance model. Health Psychol.

[37] Obradović MMS, Hamelin B, Manevski N, Couto JP, Sethi A, Coissieux MM, et al. Glucocorticoids promote breast cancer metastasis. Nature. 2019;567(7749):540–4. .10.1038/s41586-019-1019-4 10.1038/s41586-019-1019-4 30867597

[38] Arbour KC, Mezquita L, Long N, Rizvi H, Auclin E, Ni A, et al. Impact of baseline steroids on efficacy of programmed cell Death-1 and programmed death-ligand 1 blockade in patients with non-small-cell lung Cancer. J Clin Oncol. 2018;36(28):2872–8. .10.1200/JCO.2018.79.0006 10.1200/JCO.2018.79.0006 30125216

[39] Yaribeygi H, Panahi Y, Sahraei H, Johnston TP, Sahebkar A. The impact of stress on body function: a review. EXCLI J. 2017;16:1057–72. 10.17179/excli2017-480 10.17179/excli2017-480 PMC5579396 28900385

